# 10 Jahre Nationales Referenzzentrum für Hepatitis-B-Viren und Hepatitis-D-Viren in Gießen: Tätigkeiten und Erfahrungen

**DOI:** 10.1007/s00103-021-03479-7

**Published:** 2022-01-11

**Authors:** Dieter Glebe, Felix Lehmann, Nora Goldmann, Annika Giese, Yassine Hida, Wolfram H. Gerlich, John Ziebuhr, Heiko Slanina, Christian G. Schüttler

**Affiliations:** Nationales Referenzzentrum für Hepatitis-B-Viren und Hepatitis-D-Viren, Institut für Medizinische Virologie, Justus-Liebig-Universität Gießen, Schubertstr. 81, 35392 Gießen, Deutschland

**Keywords:** Akute und chronische HBV-Infektion, Akute und chronische HDV-Infektion, HBV-Immunescape-Varianten, Diagnostik der HBV- und HDV-Infektion, HBV-Resistenzmutationen, Acute and chronic HBV infection, Acute and chronic HDV infection, HBV immune escape variants, Diagnostics of HBV and HDV, HBV resistance mutations

## Abstract

Das Nationale Referenzzentrum (NRZ) für Hepatitis-B-Viren (HBV) und Hepatitis-D-Viren (HDV) befindet sich seit seiner Gründung und Berufung im Jahr 2011 am Institut für Medizinische Virologie der Justus-Liebig-Universität Gießen (JLU). In diesem Beitrag werden die Tätigkeitsbereiche des NRZ und die damit verbundenen Erfahrungen beschrieben.

Das NRZ bietet eine umfassende Beratungstätigkeit zu allen diagnostischen und klinischen Aspekten der akuten und chronischen Infektion mit HBV und HDV für den Öffentlichen Gesundheitsdienst (ÖGD), diagnostische Laboratorien, Kliniken, Forschungsinstitute und niedergelassene Ärzte. Unklare diagnostische Befunde können mit der am NRZ etablierten HBV/HDV-Spezialdiagnostik unter Verwendung von aktuellen molekularbiologischen, biochemischen und genetischen Untersuchungsmethoden analysiert, interpretiert und epidemiologische Zusammenhänge aufgeklärt werden. Das NRZ kann dabei auf eine umfangreiche Stammsammlung von vielen gut charakterisierten und klonierten HBV/HDV-Isolaten zurückgreifen, die eine vergleichende Analyse und Bewertung von antiviralen Resistenzmutationen und Immunescape-Varianten zulässt. Das NRZ initiiert und begleitet mit seinen nationalen und internationalen Partnerinstitutionen unter anderem Ringversuche zur Diagnostik der HBV-Resistenz, einschließlich Immunescape, zur Entwicklung und Validierung von internationalen Standards der Weltgesundheitsorganisation (WHO) und zur Optimierung der quantitativen HDV-Genombestimmung. Das NRZ beteiligt sich aktiv an aktuellen Empfehlungen und Leitlinien zu HBV und HDV sowie an Empfehlungen von medizinischen Fachgesellschaften. Es weist mit Beiträgen in Form von nationalen und internationalen Vorträgen sowie mit Originalarbeiten und Kommentaren in nationalen und internationalen Journalen auf aktuelle HBV/HDV-relevante Aspekte hin.

## Abstract (en)

The National Reference Center (NRC) for hepatitis B viruses (HBV) and hepatitis D viruses (HDV) has been located at the Institute of Medical Virology of the Justus Liebig University (JLU) in Giessen, Germany, since its establishment in 2011. This paper describes the NRC’s areas of activity and related experience.

The NRC offers comprehensive consulting services on all diagnostic and clinical aspects of acute and chronic HBV and HDV infections for the Public Health Service (ÖGD), diagnostic laboratories, clinics, research institutes, and physicians in private practice. Uncertain diagnostic findings can be analyzed and interpreted and epidemiological correlations clarified with the HBV/HDV special diagnostics established at the NRC using state-of-the-art molecular, biochemical, and genetic laboratory tools. The NRC has access to a strain collection of many well-characterized and cloned HBV/HDV isolates, allowing comparative analysis and evaluation of antiviral resistance mutations and immune escape variants. Together with its national and international partner institutions, the NRC initiates and supervises, among other things, interlaboratory studies for the diagnosis of HBV resistance and immune escape for the establishment and validation of international World Health Organization (WHO) standards and for the improvement of quantitative HDV genome determination. The NRC actively participates in current recommendations and guidelines on HBV and HDV and the recommendations of medical societies. It also highlights current HBV/HDV-relevant aspects with contributions in the form of national and international lectures as well as original articles and comments in national and international journals.

## Hintergrund

In Deutschland werden Nationale Referenzzentren (NRZ) und Konsiliarlabore (KL) zum Infektionsschutz und zur Überwachung gesundheitsrelevanter Infektionserreger seit dem Jahr 1995 vom Robert Koch-Institut (RKI) im Auftrag des Bundesgesundheitsministeriums (BMG) berufen [1]. Die im Jahr 2021 am RKI gelisteten 20 NRZ und 37 KL sind jeweils speziellen Erregern oder Erregergruppen zugeordnet und repräsentieren die erregerspezifische Expertise der mit diesen Funktionen beauftragten Institutionen (siehe RKI-Liste der NRZ und KL, [2]). Das Nationale Referenzzentrum für Hepatitis-B-Viren und Hepatitis-D-Viren ist seit seiner Gründung im Jahr 2011 am Institut für Medizinische Virologie (Direktor: Prof. Dr. med. John Ziebuhr) der Justus-Liebig-Universität Gießen (JLU) beheimatet. Prof. Dr. rer. nat. Dieter Glebe ist Leiter des NRZ und vertritt dieses gegenüber dem RKI und dem BMG. Ärztlicher Leiter des NRZ ist Dr. med. Christian Schüttler.

Bereits seit 1995 war das Institut für Medizinische Virologie der Sitz des damaligen KL für Hepatitis-B-Viren und Hepatitis-D-Viren unter der Leitung von Prof. Dr. phil. nat. Dr. h.c. Wolfram H. Gerlich bis zu seiner Pensionierung als Institutsleiter im Jahr 2010. Aufgrund der gestiegenen Bedeutung der HBV- und HDV-Infektionen in Deutschland erfolgte 2010 die öffentliche Ausschreibung als NRZ. Mit diesem Beitrag soll über wesentliche Aspekte der nun 10-jährigen Tätigkeit und Erfahrung des NRZ zu HBV- und HDV-Infektionen berichtet werden.

## Beratungstätigkeit für den Öffentlichen Gesundheitsdienst, Laboratorien, niedergelassene Ärzte, Kliniken und Forschungsinstitute

HBV-Infektionen sind weltweit für eine erhebliche Gefährdung der individuellen Gesundheit durch infektionsassoziierte Folgeerkrankungen sowie eine hohe Sterblichkeit durch Leberzirrhose und das primäre hepatozelluläre Karzinom (HCC) verantwortlich [3]. Nach aktuellen Angaben der Weltgesundheitsorganisation (WHO) zeigten im Jahr 2019 weltweit etwa 296 Mio. Menschen serologische Anzeichen einer aktiven HBV-Infektion (HBsAg-Nachweis), bei geschätzten 1,5 Mio. Neuinfektionen allein im Jahr 2019 [3]. Deutschland gilt hierbei als Niedrigprävalenzland [4] mit (je nach untersuchter Kohorte) geschätzten 0,3–0,7 % HBV-Infizierten (HBsAg-Positiven) in der Gesamtbevölkerung [5]. Eine aktive Impfung zur Prävention der HBV-Infektion sowie antivirale Therapeutika zur Behandlung chronischer Formen der HBV-Infektion sind verfügbar. Global waren nur 10 % aller geschätzten HBV-Infektionen diagnostiziert und nur 2 % der HBV-Infizierten hatten im Jahr 2019 Zugang zu einer antiviralen Therapie. Dagegen waren 2019 weltweit 85 % aller Kinder entsprechend der WHO-Empfehlung vollständig gegen HBV geimpft (3 Impfungen; [3]).

Die Diagnostik der HBV-Infektion ist wegen des sehr variablen Verlaufs der akuten und chronischen Infektion oft sehr anspruchsvoll und die verwendeten Testverfahren sind die mit Abstand häufigsten in der Virologie und Labormedizin. Eine aktuelle Übersicht zu erregerspezifischen Charakteristika sowie praxisnahe Informationen zur Diagnostik aller viralen Hepatitiden bieten Schüttler et al. [6]. Das NRZ bietet eine qualifizierte Beratung zu allen Aspekten der HBV/HDV-Infektion im Rahmen des Aufgabenkatalogs des RKI [7]. Beratungsbedarf haben zumeist niedergelassene Labore der klinischen Chemie, Transfusionsmedizin, Mikrobiologie und Virologie, der Bereich Arbeitsmedizin, der Öffentliche Gesundheitsdienst und die Landesgesundheitsbehörden. Die Anfragen kommen mehrheitlich aus Deutschland, bisweilen aus Österreich, der Schweiz und vereinzelt aus dem Vereinigten Königreich, Irland, Frankreich und Australien. Spezielle Laboranfragen gehen vorwiegend zu unklaren serologischen Konstellationen ein, wie etwa zu isoliert reaktiven HBsAg- oder Anti-HBc-Tests. Bei vielen Laboranfragen erfolgt eine (Nach‑)Untersuchung von Proben bzw. Probenserien am NRZ. Oft ist eine nicht ausreichende Testqualität und/oder fehlerhafte Interpretation der Testdaten des Einsenders die Ursache für unklare Befundkonstellationen.

Obwohl das NRZ im Rahmen des Aufgabenkatalogs für NRZ keine Beratungstätigkeit für Privatpersonen durchführt, werden pro Jahr etwa 800 Anfragen von Personen aus speziellen Risikogruppen oder von HBV-(/HDV-)infizierten Patienten entgegengenommen. In den Jahren 2014 und 2015 erreichten das NRZ vermehrt Anfragen zum Risiko der Übertragung von HBV in Gemeinschaftsunterkünften sowie zum Umgang mit hochvirämisch HBV-infizierten Kindern in Krippen und Kindergärten.

## Die Bedeutung der HBV-Genotypen für Diagnostik und Klinik der HBV-Infektion

HBV lässt sich nach der Sequenzvariabilität seines DNA-Genoms in mindestens 9 unterschiedliche Genotypen (Gt) A–I und Subgenotypen unterteilen [8], deren geografische Verbreitung in unterschiedlichen Regionen der Welt variiert [9]. Eine HBV-Genotypisierung wird im klinischen Alltag selten durchgeführt, kann aber entscheidende Hinweise zur Epidemiologie und Klinik der HBV-Infektion liefern [8, 10]:

1. zum Übertragungsweg (Gt B und C treten gehäuft bei Mutter-Kind-Übertragungen auf),
2. zum natürlichen Verlauf der Infektion (spontane HBeAg-Serokonversionsraten treten bei Gt A, B und D gegenüber dem Gt C zeitlich früher und gehäufter auf),
3. zur Schwere der möglichen Leberschädigung bei chronischem Infektionsverlauf bis hin zum Risiko der Entstehung eines HCC (die genannten Risiken sind bei Infektionen mit Gt C gegenüber anderen HBV-Gt deutlich erhöht; [11]) und
4. zum Ansprechen auf eine Interferontherapie. Dies ist bei chronischen HBV-Patienten bei Gt A oder B besser als bei solchen mit Gt C oder D [12].

Für das Ansprechen auf eine antivirale Therapie mit Nukleos(t)id-Analoga (NA, Inhibitoren der Reverse-Transkriptase-Domäne der HBV-Polymerase) wurden bislang keine signifikanten Unterschiede der einzelnen Genotypen gefunden [13]. Neben den jeweiligen HBV-Wildtypen sind es HBV-Varianten mit spezifischen Mutationen im viralen Genom, die im klinischen Alltag zu Therapieversagen sowie diagnostischem oder immunologischem Nichterkennen (Escape) führen können [14].

**Figure 1:**
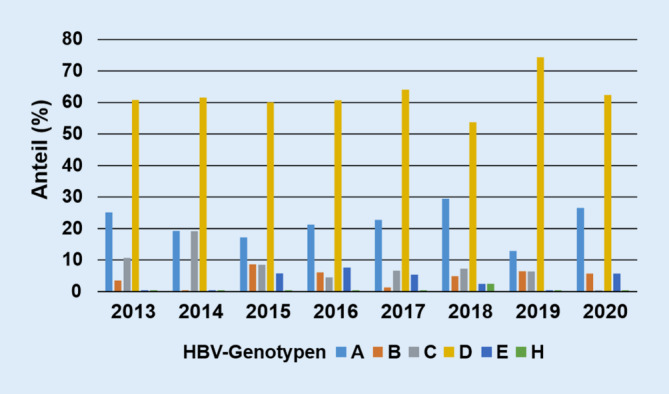

Die HBV-Genotypisierung durch HBV-DNA-Sequenzanalyse [15] wurde am NRZ auf Anfrage in den vergangenen Jahren in 355 Fällen durchgeführt (Abb. 1). Hierbei wurden spezielle Untersuchungen zum Nachweis von resistenzvermittelnden Mutationen gegenüber einer Therapie mit Nukleos(t)id-Analoga und/oder von HBsAg-Escape-Mutanten angefordert. Die am NRZ ermittelten HBV-Genomsequenzdaten wurden mit der *geno2pheno*-HBV-Plattform^1^ abgeglichen. Mit dieser Internetpräsenz, aber auch mit der vergleichbaren Plattform *HIV-Grade*^2^ können aus infizierten Patientenproben isolierte DNA-Sequenzdaten der HBV-Polymerase auf ihr potenzielles Resistenzmuster gegenüber klinisch verwendeten antiviralen Substanzen verglichen werden. Bei ausreichender Länge des *online* übermittelten HBV-Genombereichs können, aufgrund der speziellen genomischen Überlappung viraler Genbereiche für die Polymerase und die Oberflächenproteine des HBV, potenzielle Immunescape-Mutationen und der HBV-(Sub)Genotyp ermittelt werden. Die am NRZ ermittelte HBV-(Sub)Genotypverteilung (Abb. 1) ist typisch für Mitteleuropa [16].

## Virale Varianten mit Resistenz- und Immunescape-Eigenschaften

Bekannte virale Mutationen innerhalb der Reverse-Transkriptase-Domäne der HBV-Polymerase, die mit phänotypischer Resistenz gegenüber antiviralen Therapeutika assoziiert sind [17], konnten stets bei einem variablen Teil (im Mittel ca. 10 %) der untersuchten Isolate festgestellt werden. Dabei handelte es sich um Mutationen, die eine klinische Resistenz gegenüber den Wirkstoffen Lamivudin, Telbivudin, Entecavir und Adefovir vermitteln können. Genotypische Resistenzmutationen gegenüber dem Wirkstoff Tenofovir sind vergleichbar mit denen gegenüber Adefovir, jedoch führen die bislang gefundenen Mutationen in den zirkulierenden HBV-Genomen nicht zu einer klinisch relevanten Resistenz unter Therapie mit verschiedenen Prodrugs des Wirkstoffs Tenofovir im klinischen Alltag [18, 19]. Zur phänotypischen Charakterisierung und Validierung von bekannten und neu auftretenden, potenziellen Resistenzmutationen verfügt das NRZ über spezielle Inhousetests, mit denen die Resistenzprofile von HBV-Varianten phänotypisch in der Zellkultur quantitativ bestimmt werden können [17, 20, 21].

In etwa 15 % der isolierten Proben konnten klassische HBsAg-Immunescape-Mutationen charakterisiert werden. Gehäuft wurde die Immunescape-Variante G145R gefunden, die oft bei Infektionen von Neugeborenen während der perinatalen Mutter-Kind-Übertragung trotz rechtzeitig erfolgter aktiver/passiver HBV-Impfung des Neugeborenen auftritt.

Zusammenfassend liefern die Bestimmung des HBV-Genotyps und die Charakterisierung von Virusvarianten wichtige Informationen zur Epidemiologie, Diagnostik, Klinik und Therapie der HBV-Infektion und sie sind ein bedeutender Baustein der Spezialdiagnostik des NRZ. Wünschenswert wäre eine weiterführende Analytik mit der Sequenzierung möglichst aller am NRZ eintreffenden HBV-haltigen Proben mit einer Vollgenomtiefensequenzierung (Deep Sequencing) der einzelnen HBV-Genome, um weitere klinisch relevante Marker (z. B. Prä-Core-Mutationen) und eine eindeutige Subgenotypisierung zu erhalten. Für eine aktuelle deutschlandweite Übersicht des HBV-Infektionsgeschehens wäre es allerdings notwendig, dass dem NRZ Probenmaterialien möglichst aller in Deutschland neu diagnostizierten HBV-Fälle zur Verfügung gestellt würden (ca. 6800 im Jahr 2020).

## Beteiligung an der Erstellung von Leitlinien und Empfehlungen

Das NRZ war an der Erstellung der aktuellen AWMF-S3-Leitlinie zur Prophylaxe, Diagnostik und Therapie der Hepatitis-B-Virusinfektion beteiligt [22], insbesondere in den Bereichen Diagnostik (AG1) und Immunprophylaxe (AG5). Hierbei wirkte das NRZ an Empfehlungen zur Impfung von Neugeborenen mit. Eine aktive und passive Impfung des Neugeborenen innerhalb von 12 h nach der Geburt verhindert in der Regel eine HBV-Infektion des Neugeborenen bei geburtlicher Exposition mit dem Blut einer HBsAg-positiven, HBV-infizierten Mutter [23]. Mit den derzeit verfügbaren HBV-Impfstoffen ist ein Versagen der Postexpositionsprophylaxe beim Neugeborenen möglich, wenn bei Schwangeren während der Geburt sehr hohe HBV-Konzentrationen im Blut nachweisbar sind (über 2 × 10^5^ IU/ml, d. h. über 1 × 10^6^ Genome HBV/ml; [24]). Das Risiko einer Infektion beim Neugeborenen trotz erfolgter Impfung erhöhte sich dabei mit steigender HBV-Konzentration im Blut der Schwangeren (über 10^7^–10^8^ IU/ml) je nach Kohorte auf 10 % [23, 24].

Eine intensive Senkung der HBV-Konzentration der Schwangeren durch eine konsequente antivirale Therapie bis zum Geburtstermin kann dieses Risiko minimieren, da unter einer mütterlichen Virämie von 2 × 10^5^ IU/ml keine Evidenz für einen HBV-Impfdurchbruch bei aktiv und passiv geimpften Neugeborenen besteht [24]. Aktuelle internationale Leitlinien (WHO, European Association for the Study of the Liver – EASL, American Association for the Study of the Liver – AASLD) empfehlen in diesen Fällen eine antivirale Therapie der Schwangeren, bevorzugt mit dem Nukleotidanalogon Tenofovir (TDF; [22]). Die antivirale Therapie sollte möglichst früh in der Schwangerschaft begonnen werden (idealerweise nach dem 1. Trimenon, zumindest vor der 28.–32. Schwangerschaftswoche, SSW; [22, 25]), da der therapiebedingte Abfall der HBV-Konzentration im Serum der Infizierten über mehrere Log-Stufen in der Regel mehrere Monate erfordert. Gemäß den aktuellen Mutterschaftsrichtlinien wird in Deutschland die Untersuchung der Schwangeren auf eine HBV-Infektion (HBsAg-Screening) nach der 32. SSW durchgeführt. Eine entsprechende antivirale Therapie der HBV-infizierten Schwangeren kann damit erst nach diesem Termin eingeleitet werden. Eine Testung der Schwangeren in der 12. SSW oder früher wäre für einen optimalen Beginn der HBV-Therapie hilfreich und sollte nach den Empfehlungen der aktuellen S3-Leitlinien erwogen werden [22].

## Beteiligung an Empfehlungen zur beruflichen Tätigkeitseinschränkungen von chronisch HBV-Infizierten im Gesundheitswesen

In Zusammenarbeit mit der *Deutschen Vereinigung zur Bekämpfung der Viruskrankheiten (DVV) e.* *V.* wurden die *Empfehlungen zur Prävention von HBV- und HCV-Übertragungen durch im Gesundheitswesen Tätige* (Healthcare Worker, HCW) überarbeitet und aktualisiert [26]. Nosokomiale HBV-Infektionen bei Patienten durch chronisch HBV-infiziertes medizinisches Personal wurden in der Vergangenheit überwiegend bei besonders übertragungsträchtigen Tätigkeiten mit hohem Verletzungspotenzial festgestellt, wie etwa in der Thorax- und Kieferchirurgie sowie der operativen Gynäkologie. Neben der übertragungsträchtigen Tätigkeit spielte die HBV-Genomkonzentration im Blut des behandelnden HBV-infizierten HCW eine entscheidende Rolle bei der Übertragung. Bei HBV-Konzentrationen von unter 200 IU/ml sind bislang keine Übertragungen von HBV-infizierten HCW auf ihre Patienten ermittelt worden, daher entfallen besondere Tätigkeitseinschränkungen und über die Routine hinausgehende spezielle Sicherheitsmaßnahmen. Mögliche Änderungen der HBV-DNA-Konzentration im Blut sollen jedoch durch Verlaufskontrollen alle 3 Monate erfasst werden. Keine übertragungsträchtige Tätigkeit soll bei Konzentrationen über 20.000 IU/ml HBV-DNA ausgeübt werden. Bei Werten von 200–20.000 IU/ml HBV-DNA soll die berufliche Tätigkeitsausübung je nach Übertragungsgefahr individuell ermittelt werden (z. B. durch Einsetzen einer lokalen Ad-hoc-Kommission) und eventuell spezielle Sicherheitsmaßnahmen ergriffen werden.

Die hier genannten Empfehlungen wurden in der DVV-Empfehlung weiter ausgeführt [26] und sind in die aktuelle S3-Leitlinie zur Prophylaxe, Diagnostik und Therapie der Hepatitis-B-Virusinfektion eingeflossen [22].

## Erstellung und Validierung von diagnostischen Standards und Initiierung von Ringversuchen zur HBV-Resistenztestung und Immunescape

Die spezifisch antivirale Therapie der chronischen HBV-Infektion mit oral applizierten Nukleos(t)id-Analoga (NA) ist eine verträgliche und in der Regel sehr gut wirksame Therapie der chronischen HBV-Infektion [27]. Aufgrund der selbst bei Verwendung von hochpotenten Wirkstoffen (Entecavir, ETV oder Tenofovir, TDF) vergleichsweise niedrigen Ausheilungsraten (HBsAg-Verlust nach 5 Jahren) von 5 % (ETV) oder 9,8 % (TDF) muss die Therapie oft jahrelang durchgeführt werden [22]. Hierbei können bei Verwendung von NA mit niedriger genetischer Barriere (z. B. Lamivudin) antivirale Resistenzmutationen selektioniert werden, die zur verminderten Wirksamkeit oder vollständigen Resistenz der selektionierten Virusstämme gegenüber dem eingesetzten NA führen können. Insbesondere beim Vorliegen einer vollständigen Lamivudin-Resistenz kann die Wirksamkeit von Entecavir eingeschränkt sein. Neben der Kontrolle der Compliance des Patienten [28] ist auch die Bestimmung genotypischer Resistenzmutationen im Genom der zirkulierenden Virusstämme von Bedeutung, z. B. bei einem klinischen Nichtansprechen eines NA, zur Therapieplanung vor einem Wechsel des Wirkstoffs oder bei unklaren Vortherapien.

Zur Validierung und Standardisierung der diagnostischen HBV-Resistenztestung unter antiviraler Therapie und HBV-Genotypisierung hat das NRZ zusammen mit Prof. Dr. Heinz Zeichhardt und INSTAND e. V. seit 2016 erste deutschlandweite Ringversuche initiiert. Hierbei wurden auf Vorschlag des NRZ gut charakterisierte HBV-Genome aus der Genombank des NRZ mit charakteristischen Resistenzmutationen gegen weltweit verwendete NA (Lamivudin, Telbivudin, Entecavir, Adefovir, Tenofovir) ausgewählt und über INSTAND an die teilnehmenden Diagnostiklaboratorien versendet. Die Bestimmung von antiviralen HBV-Resistenzmutationen und des HBV-Genotyps wurde von den teilnehmenden Laboren mit großem Erfolg durchgeführt und soll fortgeführt werden.

## Charakterisierung und Validierung des 3. Internationalen WHO-Standards für HBsAg

Die Bestimmung des HBsAg ist neben dem Anti-HBc einer der Grundpfeiler in der Diagnostik einer HBV-Infektion. Ferner kann die Quantifizierung des HBsAg auch zur Verlaufskontrolle der HBV-Infektion und zur langfristigen Überwachung der antiviralen Therapie von chronisch HBV-Infizierten eingesetzt werden [22, 29]. Zur verlässlichen Validierung der diagnostischen HBsAg-Tests sind gut charakterisierte internationale Standards (IS) in Form von gut charakterisierten biologischen Präparaten essenziell. Das Ausgangsmaterial dieser IS-Präparate ist aber nur in begrenztem Umfang verfügbar, da es nicht aus HBsAg-produzierenden Zellkulturen stammt, sondern weiterhin aus gepoolten Plasmen von HBV-infizierten Patienten.

Das NRZ hat in enger Kooperation mit dem Paul-Ehrlich-Institut (PEI) und dem National Institute for Biological Standards and Control (NIBSC) im Vereinigtem Königreich einen neuen Internationalen WHO-Standard für HBsAg charakterisiert, der als 3. IS für HBsAg den damals fast aufgebrauchten 2. IS ersetzen sollte. Bei dem Ausgangsmaterial handelte es sich um eine nicht mehr benötigte Charge eines HBV-Vakzins aus gepoolten Plasmen von vietnamesischen Patienten mit chronischer HBV-Infektion. Das NRZ hat das gereinigte und inaktivierte HBsAg-Ausgangsmaterial sowie bereits lyophilisierte Standards auf die Eignung als HBsAg-Standard molekulargenetisch und biochemisch untersucht.

Die Sequenzierung der HBV-DNA ergab, dass es sich um ein Gemisch aus mindestens 2 HBV-Stämmen mit dem für Vietnam typischen Subgenotyp B4, jedoch genauer um 2 HBsAg-Subtypen (ayw1 und adw2) handelte [30]. Das HBsAg war produktionsbedingt zum Teil aggregiert, was eine etwas verminderte Reaktivität in diagnostischen Tests bewirkte, und es enthielt nur sehr geringe Mengen an PräS-Domänen der HBV-Oberflächenproteine [30]. Unter Federführung des NIBSC, des PEI und des NRZ konnten in einem internationalen Ringversuch die Reaktivität, Stabilität und damit die Eignung der untersuchten Charge als neuer 3. IS auch im direkten Vergleich mit dem vorherigen 2. IS bestätigt werden [31].

## HDV-Genotypenpanel zur Standardisierung der HDV-RNA-Genombestimmung

Eine akute oder aktive chronische Infektion mit dem Hepatitis-D-Virus (HDV) tritt im klinischen Alltag nur zusammen mit einer aktiven HBV-Infektion auf. Nach neuesten Berechnungen aus dem Jahr 2020 zeigen weltweit geschätzt 4,5 % aller HBV-Infizierten (HBsAg-positiv) serologische Marker einer HDV-Infektion [32]. Das zirkuläre HDV-RNA-Genom (ca. 1700 Nukleotide) zeichnet sich durch seine hohe genetische Variabilität von bis zu 40 % zwischen einzelnen Isolaten aus. Es lassen sich mindestens 8 Genotypen (Gt) und diverse Subgenotypen unterscheiden [33], die eine charakteristische geografische Verteilung aufweisen.

Der Gt 1 ist weltweit verbreitet und wird in fast 90 % aller bislang publizierten Isolaten gefunden. Der Gt 2 findet sich hauptsächlich in Asien, während der Gt 4 in Japan und China zirkuliert. Der Gt 3 ist in Südamerika vorherrschend. Die Gt 5–8 wurden bislang hauptsächlich in Afrika gefunden. Vergleichbar mit den HBV-Genotypen zeigen auch die HDV-Genotypen unterschiedliche Charakteristika im natürlichen Infektionsverlauf und in der Klinik [34].

Die diagnostische Bestimmung und Quantifizierung des HDV-RNA-Genoms erfolgt mittels Nukleinsäureamplifikationstechniken (NAT), meist in Form von spezifischen Reverse-Transkriptase-Polymerasekettenreaktionen (RT-PCR). Der Etablierung standardisierter NAT für alle HDV-Genotypen stehen 3 wesentliche genetische Besonderheiten des HDV-Genoms entgegen [33]: (1) die hohe genetische Variabilität der einzelnen HDV-Genotypen und Varianten, (2) der hohe Gehalt an Cytosin und Guanin (CG-Gehalt) sowie (3) die zahlreichen intragenomischen Basenpaarungen der RNA, die bis zu 70 % des gesamten HDV-Genoms umfassen können. Dies führte in der Vergangenheit dazu, dass es in vielen kommerziellen und Inhouse-RT-PCR-Assays zu Unterquantifizierungen kam oder falsch-negative Ergebnisse erzielt wurden [35], insbesondere bei der Analyse der HDV-Gt 5–8 [36]. Seit 2013 ist ein erstes internationales WHO-HDV-Standardplasma des Genotyps 1 verfügbar [37]. Zur Validierung der HDV-RT-PCR wurde am NRZ ein Panel relevanter HDV-Vollgenome der HDV-Gt 1–8 synthetisiert (unter Nutzung von in öffentlichen Datenbanken publizierten Sequenzen) und in Form von komplementärer DNA (cDNA) als replikationsfähige HDV-Genome in Plasmide kloniert. Die Produktion und Sekretion von HDV-Partikeln erfolgten im Labor in Leberzellkulturen nach Kotransfektion der jeweiligen HDV-Plasmidklone mit einem Plasmid, das selektiv die 3 Oberflächenproteine des HBV exprimiert. Die in den Überstand der Zellkulturen sekretierten HDV-Partikel wurden gereinigt, quantifiziert und das Infektions- und Replikationspotenzial der so erhaltenen HDV-Präparate in der Zellkultur mit HDV-suszeptiblen humanen Leberzellkulturen überprüft. Eine Quantifizierung dieses HDV-Genotypenpanels erfolgte nach RNA-Extraktion mit der am NRZ verwendeten Inhouse-HDV-RT-PCR. Als Vergleich diente der WHO-Standard des Gt 1.

**Figure 2:**
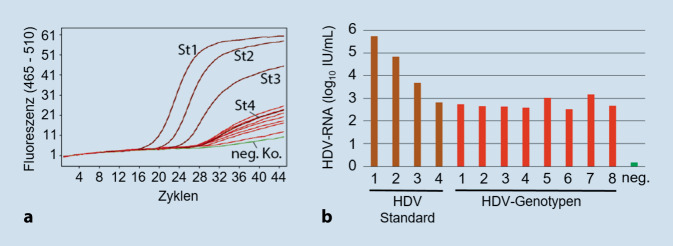

Das Ergebnis der Quantifizierung der einzelnen HDV-Präparate aus der Zellkultur zeigte eine vergleichbar gute Quantifizierung der Gt 1–8 gegenüber dem WHO-Standard, des Gt 1 auch bei den hier gewählten niedrigen Konzentrationen an HDV-RNA von 5 × 10^2^ IU/ml (Abb. 2). Eine CE-IVD-zertifizierte, kommerziell erhältliche HDV-RNA-NAT konnte unter identischen Testbedingungen ebenfalls alle HDV-Genotypen erkennen. Eine weitere quantitative HDV-RT-PCR, jedoch mit anderen Testbedingungen, wurde in Zusammenarbeit mit Kolleginnen und Kollegen des Uniklinikums Hamburg-Eppendorf mit einer vollautomatisierten PCR-Plattform etabliert [38].

Analysen in der Zellkultur haben kürzlich gezeigt, dass das HDV zur Umhüllung und Ausschleusung aus der Zelle auch die Oberflächenproteine des Dengue- und West-Nil-Virus und des Hepatitis-C-Virus (HCV) benutzen kann [39]. Experimente in geeigneten Tiermodellen zeigten, dass eine zugrunde liegende Infektion mit HCV eine Infektion und Ausbreitung von HDV innerhalb der Leber auch in Abwesenheit einer HBV-Infektion unterstützt [39]. Klinisch wurde die Anwesenheit von HDV-RNA bei HCV-infizierten Patienten in Abwesenheit von serologischen Markern einer HBV-Infektion in einer kleinen Studie (*N* = 160) in Venezuela bislang nur bei einem einzigen Patienten gefunden [40]. Bei 2 weiteren HCV-Patienten ohne HBV-Marker konnten Antikörper gegen HDV nachgewiesen werden. Aktuelle Studien aus Deutschland ([38]; *N* = 323), Frankreich ([41]; *N* = 2123) und China/Deutschland ([42]; *N* = 5080) konnten diese Befunde nicht bestätigen.

Das HDV des Menschen war bislang der einzige Vertreter der Gattung *Deltavirus*. HDV-ähnliche RNA-Sequenzen wurden kürzlich auch in weiteren Vertebraten und Invertebraten entdeckt [43]. Das NRZ war im Jahr 2020 an der Entdeckung und Charakterisierung des ersten HDV-ähnlichen Virus aus einer Säugetierspezies beteiligt, die nicht zur Familie der Primaten gehört (Stachelratte, *Proechimys semispinosus;* [44]). Deren virale RNA zeigte starke genomische Ähnlichkeiten zum HDV des Menschen und konnte in humanen Leberzellen in der Zellkultur autonom replizieren. Die Untersuchung von HDV-ähnlichen Viren kann wichtige Hinweise zum phylogenetischen Ursprung des HDV liefern; die bisher charakterisierten Isolate scheinen jedoch keine Bedeutung für den klinischen Alltag der HDV-Infektion zu haben.

## Nationale und internationale Kooperationen sowie Bereitstellung spezieller Methoden

Im Jahr 2016 hat die WHO die globale Elimination der viralen Hepatitiden als öffentliche Gesundheitsgefährdung beschlossen und 2021 erneuert [3]. Auf Einladung des RKI hat das NRZ im Jahr 2019 auf einem interdisziplinären Arbeitstreffen in Berlin mögliche neue Wege zur Eliminierung der Hepatitis B, C und D in Deutschland besprochen [45]. Auf internationaler Ebene wurde dieses Ziel zusammen mit dem Expertengremium *International Coalition to Eliminate HBV (ICE-HBV)* diskutiert und verbesserte Methoden für weiterführende Untersuchungen bei HBV-Infektionen erarbeitet [46]. 2019 hat das NRZ als Mitglied eines internationalen Arbeitskreises zur okkulten HBV-Infektion (OBI) neue Empfehlungen zur Diagnose und Therapie der OBI erarbeitet [47].

Mit Prof. Dr. Joachim Geyer, JLU Gießen, wurde die HBV/HDV-Interaktion mit seinem zellulären Rezeptor NTCP in einem neuartigen HBV/HDV-suszeptiblen Zellkultursystem untersucht [48]. Dieses System wird am NRZ zur Phänotypisierung von Virusmutanten und Bestimmung von neutralisierenden (Impf)Antiseren verwendet [49] sowie zur Abschätzung des zoonotischen Potenzials neuartiger Hepadnaviren eingesetzt, die von Prof. Dr. Felix Drexler, Charité Berlin, in verschiedenen Säugetieren (Primaten [50], Fledermäusen [51], Eseln [52], Spitzmäusen [53]) identifiziert wurden. Ein neues Hepadnavirus aus einer mittelamerikanischen Fledermausart (zeltbauende Fledermaus, *Uroderma bilobatum*) zeigte ein zoonotisches Potenzial, da es humane Hepatozyten über den humanen HBV-Rezeptor *in vitro* infizieren konnte und durch Antiseren von erfolgreich HBV-geimpften Personen nicht neutralisiert wurde [51].

Die neuartigen Hepadnaviren wurden mittlerweile beim *International Committee on Taxonomy of Viruses* (ICTV) von der Arbeitsgruppe Hepadnaviren katalogisiert [54], deren Vorsitz Prof. Dr. Dieter Glebe 2021 übernommen hat. Mit PD Dr. Florian van Bömmel und Prof. Dr. Thomas Berg, Leipzig, wurden neue klinische Biomarker identifiziert, die eine erfolgreiche Therapie bei chronisch HBV-Infizierten anzeigen [55] und zur Identifizierung von „inaktiven chronischen HBV-Trägern“ beitragen können [56]. Weitere HBV-relevante Biomarker wurden in Zusammenarbeit mit Prof. Dr. Markus Cornberg und Prof. Dr. Heiner Wedemeyer, Hannover, analysiert [57, 58].

## Fazit

Aufgrund der infektionsassoziierten Folgeerkrankungen, wie Leberzirrhose und HCC, bleiben insbesondere die chronischen Formen der HBV- und HDV-Infektion weiterhin eine weltweite Bedrohung der individuellen Gesundheit. Die Diagnostik der HBV- und HDV-Infektion ist wegen des sehr variablen Verlaufs der akuten und chronischen Verlaufsformen auch mit modernsten Analysemethoden oft sehr anspruchsvoll. Das NRZ bietet seit 10 Jahren eine qualifizierte Beratung zu allen Aspekten der HBV/HDV-Infektion und beteiligt sich an der Erstellung von nationalen und internationalen Leitlinien sowie Empfehlungen zur Prävention, Diagnostik und Therapie der HBV/HDV-Infektion. Darüber hinaus sind die Entwicklung und kontinuierliche Verbesserung von diagnostischen Testverfahren (z. B. zur HDV-RNA-Diagnostik), die Initiierung von Ringversuchen und die dazugehörige Charakterisierung von internationalen diagnostischen Standardpräparaten ein wesentlicher Bestandteil der NRZ-Tätigkeiten. Im Verbund mit nationalen und internationalen Partnern charakterisiert das NRZ auch weiterhin bekannte und neu entdeckte HBV/HDV-Varianten. Es berichtet über deren Detektierbarkeit in diagnostischen Testsystemen sowie zur *In-vitro-*Infektiosität und Schutzwirkung von HBV-Impfstoffen.

## Footnotes

1 http://hbv.bioinf.mpi-inf.mpg.de.

2 www.hiv-grade.de.
